# 

**Published:** 1896-11

**Authors:** 


### CONTENTS:

### SELECTIONS.

Article I.—The Dental Filling. Joseph Head, D. D. S. - - 289

" II.—New Jersey State Dental Society. - - - 294

" III.—Roentgen Rays—Their Application To Medicine and Surgery. J. Clifford Perry, M. D. - - - 307

" IV.—On "Light." By Miles Standish, M. D. . - - 317

### MONTHLY SUMMARY.

Infiltrated Anesthesia in Dentistry. - - - - - 328

Green-stain. - - - - - - - - - - 329

Preparation of Cavities. - - - - - - - 330

Felxible Rubber Edges. - - - - - - - 331

The Soldering Iron. - - - - - - - 331

Lining for Rubber Plates. - - - - - - 332

Preparing a Tooth for Porcelain Crown. - - - - 332

Dental Pain. - - - - - - - - - 333

The Greek Surgeons. - - - - - - - 333

Resuscitation from Electrical Shock. - - - - - 334

Tin and Gold Filling. - - - - - - - 334

To Clean and Remove Stains from Teeth. - - - - 334

Local Anesthetic. - - - - - - - 335

The Saving of the Pulp. - - - - - - - 335

Treatment of Abscess. - - - - - - - 335

Long Operations. - - - - - - - - 336

The Human Voice- - - - - - - - - 336

